# Improving Compliance With the British Orthopaedic Association Standards for Trauma and Orthopaedics (BOAST) Guidelines for Early Weight Bearing Following Ankle Fracture Fixation: A Closed-Loop Audit From a UK District General Hospital

**DOI:** 10.7759/cureus.88092

**Published:** 2025-07-16

**Authors:** Ahmed Mohamed, Usman Fuad, Mohamed Zahed, Maimen Shyam, Alaa Elasad, Aqeel Aqeel

**Affiliations:** 1 Trauma and Orthopaedics, Royal Cornwall Hospital, Truro, GBR; 2 Orthopaedics, John Radcliffe Hospital, Oxford University Hospitals NHS Trust, Oxford, GBR; 3 General Practice, Zagazig University, Zagazig, EGY; 4 General Surgery, Royal Cornwall Hospital, Truro, GBR

**Keywords:** ankle fracture, ankle orif, boast guidelines, early weight bearing, orthopaedic audit

## Abstract

Background

Ankle fractures are common injuries, and in many cases, operative treatment is required. While the indications for surgical fixation are well established, the optimal timing for weight bearing after open reduction and internal fixation (ORIF) remains controversial. Some surgeons continue to favour a conservative approach involving delayed weight bearing, whereas others encourage early mobilisation. The recent guidelines recommend early weight bearing unless there is a documented concern regarding fixation stability or soft tissue condition. Adherence to these guidelines promotes patient satisfaction and reduces strain on healthcare services. This closed-loop audit aimed to compare our department's practice at Royal Cornwall Hospital against the British Orthopaedic Association Standards for Trauma and Orthopaedics (BOAST) standard for postoperative mobilisation after ankle fracture fixation.

Methods

This closed-loop audit involved both retrospective and prospective analysis of postoperative weight-bearing status in patients undergoing ORIF for isolated, closed ankle fractures at a single UK centre. Two audit cycles were performed. The first cycle (November 2023 to May 2024) retrospectively analysed patients' postoperative instructions. Following an educational intervention, including departmental emails and posters, a second cycle was prospectively conducted between July and November 2024. Inclusion criteria included skeletally mature patients (>18 years old) who underwent surgical treatment for isolated, closed ankle fractures. Exclusion criteria included open fractures, associated injuries, conservative management, or treatment with hindfoot nails.

Results

In the first cycle, 112 patients met the inclusion criteria. Of these, 56 patients (50%) were advised against early weight bearing, while 56 patients (50%) were advised to commence early weight bearing in a cast or boot. In the second cycle, 58 patients were assessed; 18 patients (31%) were advised against early weight bearing, and 40 patients (69%) were advised to commence early weight bearing, indicating significant improvement following intervention.

Conclusion

A targeted educational approach, including the distribution of BOAST guidelines and departmental discussions, led to improved compliance with early weight-bearing protocols. This audit highlights the value of team-based education and proactive audit cycles in enhancing adherence to national standards and improving service provision.

## Introduction

Ankle fractures are among the most frequently encountered orthopaedic injuries and represent one of the most common lower limb fractures. Hospital admission rates for these injuries range from 71 to 180 per 100,000 patients per year [1-4]. In a study by Scott et al., 203,261 patients in England were admitted with ankle fractures over a 10-year period, and 75% required surgical fixation via open reduction and internal fixation (ORIF) [5].

Despite the high incidence and clear surgical indications for ORIF, postoperative rehabilitation protocols remain heterogeneous, with considerable variation in weight-bearing recommendations among surgeons. Early weight bearing is typically defined as initiation within three weeks of surgery, while delayed weight bearing refers to initiation after three weeks [6].

Historically, rehabilitation protocols have favoured delayed weight bearing to reduce the risk of fixation failure and promote bone healing. However, recent national guidelines from the British Orthopaedic Association (BOA) and the British Orthopaedic Foot and Ankle Society (BOFAS), specifically the British Orthopaedic Association Standards for Trauma and Orthopaedics (BOAST) 12, recommend early weight bearing unless there are documented concerns regarding the stability of fixation or the soft tissue condition [7]. Early mobilisation is believed to promote bone healing through mechanical stimulation and to enhance functional recovery [8,9].

Despite the strength of this guidance, implementation in practice has lagged behind [10-12]. Multiple studies have demonstrated that early weight bearing leads to improved outcomes and shorter recovery periods [13,14]. Nonetheless, persistent concerns about potential fixation failure continue to deter some surgeons from adopting early mobilisation protocols. As a result, delayed or non-weight-bearing protocols are still the most common approach over the past 10 years [15]. Weight-bearing restrictions, however, affect both patient satisfaction and healthcare service significantly; patients find insignificance as delayed weight bearing increases their dependence on health and social services, increases their recovery period and delays return to work, with associated financial and psychological consequences [16-18].

This audit aims to evaluate and improve the quality of care provided to patients undergoing ankle ORIF. Specifically, this closed-loop audit assesses compliance with BOAST 12 guidelines regarding postoperative weight-bearing instructions, identifies gaps between current practice and national recommendations and implements targeted interventions. Conducted in a district general hospital in the United Kingdom, the audit further seeks to contribute to broader quality improvement efforts in line with value-based care and enhanced recovery after surgery (ERAS) principles in orthopaedics [19].

## Materials and methods

This closed-loop audit was conducted in the Trauma and Orthopaedics Department at Royal Cornwall Hospital, a district general hospital in the United Kingdom. The project was approved by the hospital's audit team (with ID 2820) and supervised by an associate specialist in trauma and orthopaedics. As a quality improvement audit using routinely collected clinical data, formal ethical approval was not required.

Inclusion criteria comprised skeletally mature patients (aged >18 years) who sustained isolated, closed ankle fractures requiring surgical intervention via open reduction and internal fixation (ORIF). Exclusion criteria included open fractures, ankle fractures associated with other injuries, skeletally immature patients, conservatively managed fractures and cases treated with hindfoot nails.

Two audit cycles were undertaken: The first cycle was conducted retrospectively from November 2023 to May 2024, and the second cycle was conducted prospectively from July to November 2024. Data were collected from the hospital's electronic medical records by reviewing postoperative notes and radiographs. Variables collected included patient age, fracture pattern (classified according to AO Foundation/Orthopaedic Trauma Association {AO/OTA} classification), surgical fixation method, the presence and management of syndesmotic injury, relevant medical comorbidities, documented postoperative weight-bearing instructions and specific documented reasons for delayed weight bearing. The documented postoperative weight-bearing instructions were evaluated against the standards set by the BOAST 12 guidelines [7].

Findings from the first audit cycle were presented at the local audit meeting at Royal Cornwall Hospital. During this meeting, several strategies were discussed to improve adherence to the BOAST guidelines. This included developing a sustainable and department-wide educational approach to raise awareness among all team members. Agreed-upon interventions included sending departmental emails, displaying posters within the orthopaedic unit and distributing the guidelines among staff.

Following the implementation of these measures, a second audit cycle was conducted. Data were collected and analysed using the same parameters and methodology as in the initial cycle. The results demonstrated a marked improvement in clinical practice, highlighting the effectiveness of education as a sustainable, low-resource and impactful tool for enhancing compliance with national standards.

Data were analysed using Python (v3.11) (Python Software Foundation, Wilmington, DE) with the SciPy statistical library and Microsoft Excel (Microsoft Corp., Redmond, WA). Categorical variables were compared between audit cycles using a chi-square test for independence. Effect sizes were calculated, including risk ratios with 95% confidence intervals (CI), absolute risk reduction and number needed to treat (NNT). Effect sizes for proportion differences were calculated using Cohen's h, where h = 2(arcsin√p1 - arcsin√p2). Cohen's h values are interpreted as small (h = 0.2), medium (h = 0.5) and large (h = 0.8) effects, providing standardised measures of the magnitude of change between audit cycles. Statistical significance was set at p < 0.05.

## Results

During the first audit cycle, we retrospectively reviewed 112 patients who underwent ankle fracture fixation between November 2023 and May 2024. Data were collected from postoperative notes and analysed using Microsoft Excel. All cases included clearly documented postoperative weight-bearing instructions. Among these patients, 37 (33%) had uni-malleolar fractures, 36 (32%) had bi-malleolar fractures and 39 (35%) had tri-malleolar fractures. A total of 43 (38%) patients had associated syndesmotic injuries. Lateral malleolar fractures were treated using either distal fibular locking plates or one-third tubular plates. Medial malleolar fractures were managed with either cannulated screws or plates, while posterior malleolar fractures were fixed using plates. Syndesmotic injuries were stabilised with either syndesmotic screws or tightrope fixation. When compared with BOAST guideline recommendations, only 56 (50%) patients were advised to commence early weight bearing. The remaining 56 (50%) patients were advised to delay weight bearing, despite the absence of documented contraindications.

Following the first cycle, findings were presented at the monthly orthopaedic audit meeting at Royal Cornwall Hospital. To improve compliance with BOAST guidelines, the following actions were taken: the dissemination of updated guidelines via departmental email to ensure staff awareness; display of educational posters in clinical areas, highlighting audit findings and BOAST recommendations; and agreement on implementing a structured rehabilitation pathway, including a plan to re-audit. These measures aimed to educate staff, standardise postoperative care and encourage early mobilisation where appropriate.

The second audit cycle prospectively reviewed 58 patients between July and November 2024 using the same methodology and inclusion criteria as the first cycle. Of these, 17 (29%) patients had uni-malleolar, 25 (43%) had bi-malleolar and 16 (28%) had tri-malleolar ankle fractures. Thirty-one (53%) patients had syndesmotic injuries, all treated with the same fixation techniques as in the initial cycle. Post-intervention analysis showed a marked improvement in adherence to BOAST guidelines: 40 (69%) patients were advised to initiate early weight bearing, while only 18 (31%) patients were advised to delay weight bearing.

**Figure 1:**
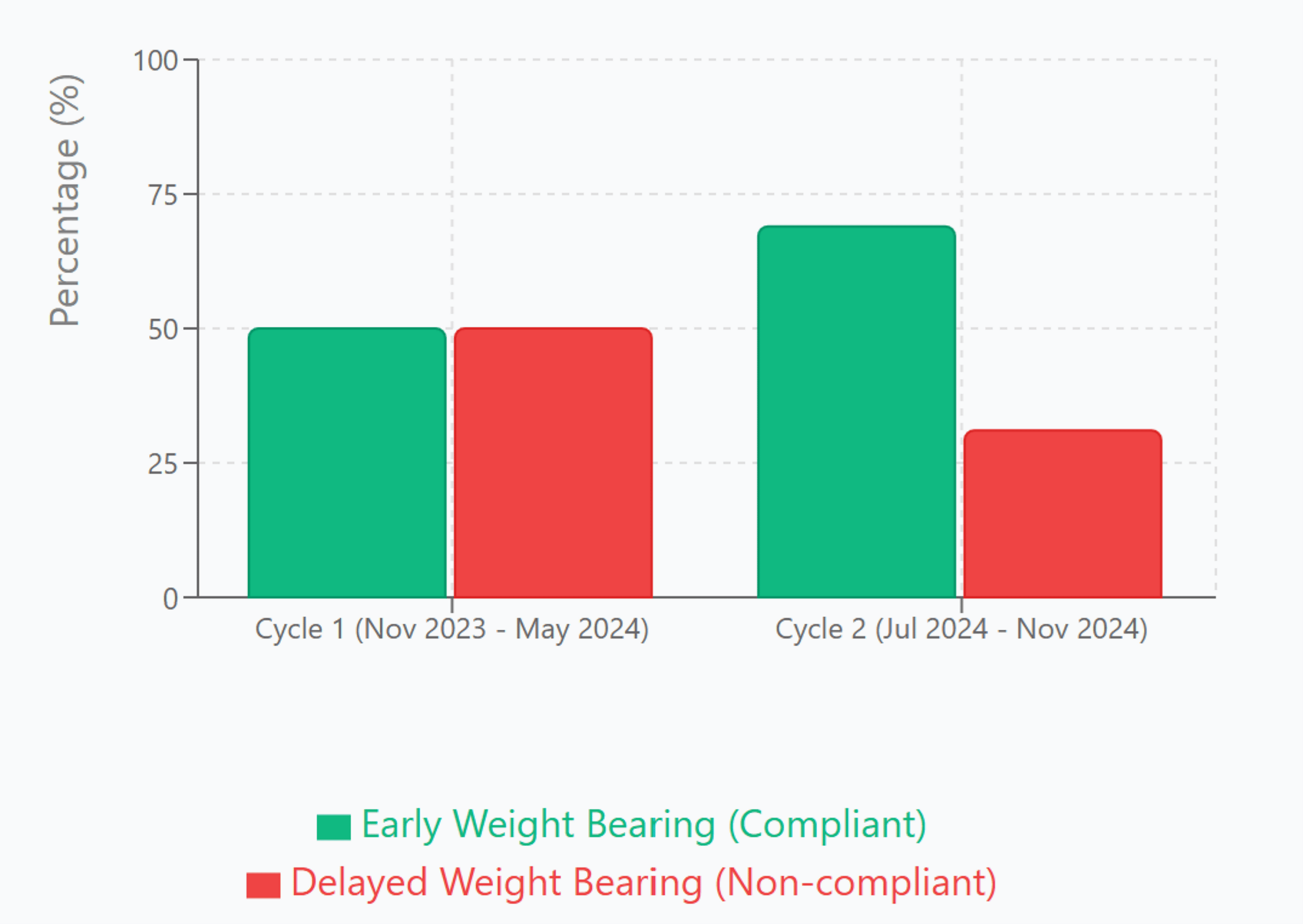
BOAST Guideline Compliance by Audit Cycle BOAST: British Orthopaedic Association Standards for Trauma and Orthopaedics

**Figure 2:**
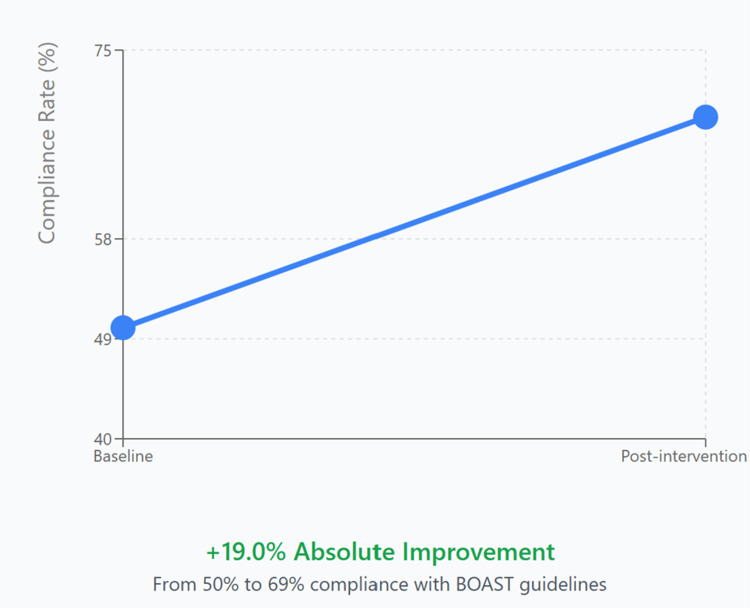
Compliance Improvement Trend BOAST: British Orthopaedic Association Standards for Trauma and Orthopaedics

The comparison of both cycles (Figure 1 and Table 1) revealed a 19% absolute improvement in compliance (95% CI: 3.9%-34.1%). The number needed to treat (NNT) was 5.3, indicating that for every five patients exposed to the intervention, one additional patient received guideline-compliant care. Full statistical outcomes are detailed in Table 2 and visualised in Figure 2.

**Table 1:** Comparison of Weight-Bearing Recommendations Between Audit Cycles Statistical analysis performed using a chi-square test for independence to compare categorical variables between audit cycles CI: confidence interval

Parameter	Cycle 1 (n = 112)	Cycle 2 (n = 58)	Difference	Chi-square value	95% CI	P-value
Early weight bearing, n (%)	56 (50.0%)	40 (69.0%)	+19.0%	5.591	3.9 to 34.1	0.018
Delayed weight bearing, n (%)	56 (50.0%)	18 (31.0%)	-19.0%	5.591	-34.1 to -3.9	0.018

**Table 2:** Statistical Analysis Results Statistical measures calculated using a chi-square test for independence, with effect sizes and confidence intervals (CI) computed using standard statistical methods

Measure	Value	95% CI	Interpretation
Chi-square (χ²)	5.591	-	p = 0.018
Risk ratio	1.61	1.12-2.32	Cycle 1 versus cycle 2 non-compliance
Absolute risk reduction	19.0%	3.9%-34.1%	Reduction in non-compliance
Number needed to treat	5.3	2.9-25.6	Patients to benefit one additional
Effect size (Cohen's h)	0.39	-	Moderate effect

## Discussion

This closed-loop audit evaluated two key outcomes. First, it assessed adherence to the BOAST 12 guidelines regarding postoperative weight-bearing status in patients who underwent open reduction and internal fixation (ORIF) for ankle fractures. Second, it highlighted the impact of departmental education and multidisciplinary discussion on aligning clinical practice with national standards.

Despite robust evidence supporting early weight bearing in appropriately stabilised ankle fractures, traditional conservative approaches remain common. Historically, many surgeons have advised patients to remain non-weight bearing for up to six weeks postoperatively to minimise the risk of non-union or fixation failure. However, evolving clinical guidelines and recent studies challenge this longstanding protocol.

The BOAST 12 guidelines, developed by the British Orthopaedic Association (BOA) and the British Orthopaedic Foot and Ankle Society (BOFAS), advocate for early weight bearing in most patients, unless there are documented concerns about fixation stability or soft tissue compromise. This approach is supported by clinical trials such as that by Smeeing et al. (2020), which demonstrated that early mobilisation improved recovery times and patient satisfaction without increasing the risk of complications [14].

However, our audit suggests that real-world practice does not always reflect this guidance. Possible reasons for this gap include the lack of awareness or the dissemination of updated protocols and surgeon preference, influenced by historical practice and perceived, but undocumented, concerns regarding fixation stability. This is consistent with findings by Fennelly et al. (2021), who reported variability in the application of BOAST guidelines across the United Kingdom, particularly regarding postoperative rehabilitation [20].

From a patient-centred perspective, the consequences of delayed mobilisation are significant. Prolonged non-weight bearing is associated with increased risk of venous thromboembolism (VTE), muscle atrophy, joint stiffness and psychological distress. It also extends dependence on healthcare and social services, delays return to work and reduces overall patient satisfaction [16,18].

Following the first audit cycle, our department implemented a series of low-cost, high-impact interventions, including educational posters, email reminders and departmental discussions. These efforts led to a measurable improvement in compliance with BOAST guidelines. Such interventions are easily replicable and sustainable, particularly if incorporated into junior doctor inductions and departmental standard operating procedures.

Finally, this audit demonstrates the practical value of the Plan-Do-Study-Act (PDSA) model in clinical settings. By identifying a gap, introducing a targeted intervention and reassessing outcomes, we not only improved clinical practice but also strengthened the culture of reflective, evidence-based care within our department.

Study limitations

This audit has several limitations that warrant consideration. As a single-centre study, generalisability to other institutions may be limited. The relatively small sample size in the second cycle (n = 58) compared to the first cycle (n = 112) may affect the robustness of statistical comparisons. We did not assess patient-reported outcomes or functional recovery measures, which would provide additional insight into clinical effectiveness.

Future directions

Building on these findings, future initiatives should focus on embedding early weight-bearing protocols into standardised care pathways and junior doctor orientation programmes. Long-term follow-up studies examining patient-reported outcomes and functional recovery would strengthen the evidence base for these quality improvement efforts. The integration of decision-support tools within electronic health records could further enhance compliance by providing real-time guidance at the point of care. Additionally, expanding this audit to multiple centres would provide valuable insights into implementation strategies across different organisational contexts.

## Conclusions

Although BOAST 12 guidelines clearly advocate for early weight bearing following ankle ORIF, our audit highlights persistent variability in clinical practice among surgeons. These discrepancies suggest that the real-world application of national standards remains inconsistent, often influenced by individual preferences or outdated rehabilitation practices.

However, our findings also demonstrate that simple, low-cost interventions, such as staff education, departmental discussion and guideline dissemination, can significantly improve adherence to evidence-based protocols. By fostering a shared understanding of current guidelines and embedding them into routine practice, departments can enhance the quality, consistency and efficiency of patient care. This audit reinforces the importance of continuous quality improvement and supports the role of targeted education in driving clinical change. As healthcare systems increasingly emphasise value-based care and enhanced recovery, aligning local practice with national standards such as BOAST 12 should be a priority across orthopaedic units.
